# Direct agglutination test: Evolution and significance as a simple and safe alternative to tissue aspiration procedures in the diagnosis of visceral leishmaniasis

**DOI:** 10.1371/journal.pone.0319118

**Published:** 2025-06-24

**Authors:** Abdallah el Harith, Hussam Ali Osman, Durria Mansour, Saul J. Semiao-Santos, Elfadil Abass

**Affiliations:** 1 School of Pharmacy, Ahfad University for Women, Omdurman, Sudan; 2 Department of Medical Laboratory Science, Faculty of Medical and Health Sciences, Liwa University, Abu Dhabi, United Arab Emirates; 3 Durria Mansour, Department of Research and Grants Unit, Ahfad University for Women, Omdurman, Sudan; 4 Agrupamento de Ciências Naturais da Escola Básica de Santo António, Parede, Lisboa, Portugal; 5 Department of Clinical Laboratory Science, College of Applied Medical Sciences, Imam Abdulrahman Bin Faisal University, Dammam, Saudi Arabia; World Health Organization, Regional Office for South-East Asia, INDIA

## Abstract

Although tissue aspiration procedures (TAPs) are considered the gold standard for visceral leishmaniasis (VL) diagnosis, they often fail to detect disease progression before the amastigote-demonstrable phase. To overcome this limitation, a primary direct agglutination test (DAT) was developed using intact *Leishmania donovani* promastigotes initially treated with trypsin as the antigen. To enhance the exposure of specific epitopes on the promastigote surface and antibody-binding sites, alternative proteolytic agents were evaluated and incorporated into antigen processing and test execution. This approach led to significant or complete inhibition of agglutination in most known cross-reacting disorders. The LQ-DAT consistently demonstrated highly reproducible results across diverse geographical regions, regardless of the *L. donovani* sub-species or strain. To facilitate routine implementation and local production, the LQ-DAT processing know-how was disseminated to all major VL-endemic areas. Sensitivities comparable to TAP outcomes were demonstrated in 2,224 of 2,697 VL cases successfully diagnosed and treated over 35 years of routine, epidemic, and outbreak evaluations. Notably, 473 (17.5%) of these cases were symptomatic, with TAP-negative but LQ-DAT-positive results, and responded favorably (98.0%−100%) to specific treatment. Given the lower sensitivity also demonstrated by LQ-DAT, TAP does not meet the criteria for classification as the gold-standard VL diagnostic. Consequently, a positive response to specific anti-leishmanial treatment is recommended as a benchmark for diagnostic reliability. Beyond its advantage in detecting VL at earlier stages compared to TAP, the improved LQ-DAT described here also exhibited feasibility and stability required for local production in low-resource settings.

## Introduction

Due to the well-documented poor sensitivity and health risks associated with tissue aspiration procedures (TAPs), the World Health Organization (WHO) recommended the development of “simple and reliable immune-epidemiological techniques for the specific diagnosis of leishmaniasis” (WHO, TDR/Leish-SWG (3)/83.3).

Although spleen aspiration is the most sensitive of the TAPs, it still falls short of achieving 100% sensitivity for the diagnosis of visceral leishmaniasis [1–3]. The necessity for precautionary measures to prevent internal bleeding has further rendered this procedure impractical for routine or large-scale application. Its execution requires specialized skills and expertise, often unavailable in rural or medium-sized hospitals. Additionally, successful spleen aspiration depends on the precise differentiation of *L. donovani* amastigotes from other intracellular microorganisms.

Bone marrow aspiration, while relatively less risky, remains a painful procedure. When conducted under poor hygienic conditions, it poses a risk of septicemia. Moreover, its sensitivity is lower compared to spleen aspiration [4–6].

Lymph node aspiration, being significantly less invasive and more feasible in rural health settings, is widely performed in most VL-endemic areas [7,8]. However, even when combined with advanced molecular techniques such as PCR, it fails to reliably detect early or mild VL infections [7].

Non-invasive classical sero-diagnostic methods, such as the indirect immunofluorescence test (IFAT), enzyme-linked immunosorbent assay (ELISA), or complement fixation test (CFT), have not provided sufficient justification for initiating or withholding treatment. Although counterimmunoelectrophoresis (CIE) demonstrated superiority over commercial indirect hemagglutination (IHA), it was primarily effective in identifying cases that also tested positive with TAPs [9–13]. Despite improvements in methodologies, including the incorporation of purified or cloned immune reactants to detect antibodies or antigens, no significant breakthroughs in VL diagnosis were achieved using these or similar classical serodiagnostic methods [14,15].

Significant progress in VL diagnosis was made with the introduction of immunochromatographic techniques. A rapid strip test, utilizing recombinant antigen (K39) demonstrated adequate reliability and ease of application [16]. Using the same format, other recombinant antigens (K9, K26, and K28) were evaluated to enhance sensitivity [17]. Despite the high reliability reported for these rapid tests, as well as the recently developed loop-mediated isothermal amplification (LAMP) technique in confirmed VL cases, more evaluation is required to determine their sensitivity for detecting VL prior to the parasite-demonstrable phase (early VL) [17]. To achieve this, conventional and quantitative polymerase chain reaction (PCRc and qPCR) techniques were introduced.

However, due to the high technical demands of molecular tools, they have also failed to meet the criteria for gold-standard diagnostics for VL. Limitations such as inadequate laboratory infrastructure, difficulties in obtaining essential reagents, and a lack of specialized expertise have hindered their implementation in resource-limited areas such as Sudan [17–19].

Despite being largely replaced, direct agglutination techniques, which rely on the specific binding of pathogen surface antigens to corresponding antibodies, remain viable candidates for optimization as practical diagnostic tools. Beyond VL, macro- and micro-direct agglutination test formats have been applied with varying success to diagnose African trypanosomiasis (AT) and American trypanosomiasis (Chagas’ disease, CD) [20–22]. A simple, instantly readable macro-agglutination test using plastic-coated cards was also introduced for AT detection [22]. While easy to use, several challenges emerged during antigen processing and test execution. Specifically, identifying and isolating the target *Trypanosoma* species surface antigen variant and determining the reaction endpoint in AT and VL complicated standardization and reproducibility [23].

Primarily due to its high potential for local production in resource-limited areas, compared to the plastic-coated card format, efforts were focused on optimizing the V-shaped microtiter agglutination setup to enhance VL diagnostic performance.

Although this report does not present novel findings, it compiles, integrates, and summarizes results from studies conducted by our group and collaborators over the past 35 years on optimizing, and evaluating the liquid direct agglutination test (LQ-DAT)) as an alternative to invasive tissue aspiration procedures (TAPs) in VL diagnosis. With an additional advantage for local production if also compared with the current molecular tools, LQ-DAT is proposed as sustainable, readily available biomonitoring tool to support routine surveillance in VL-endemic, resource-limited regions.

## Materials and methods

**Ethical Considerations:** The blood, serum, and organ aspirate samples from humans and animals used in this study were obtained from previously conducted research that had been reviewed and approved by institutional ethics committees in the respective countries, as referenced [23–31,34–46,50,57,67]. All human samples included in this study were collected with the informed consent of patients or their guardians. Additionally, all human samples were anonymized prior to testing and inclusion in this study. The scientific use of these samples was authorized by local authorities in the respective countries.

**Fig 1:**
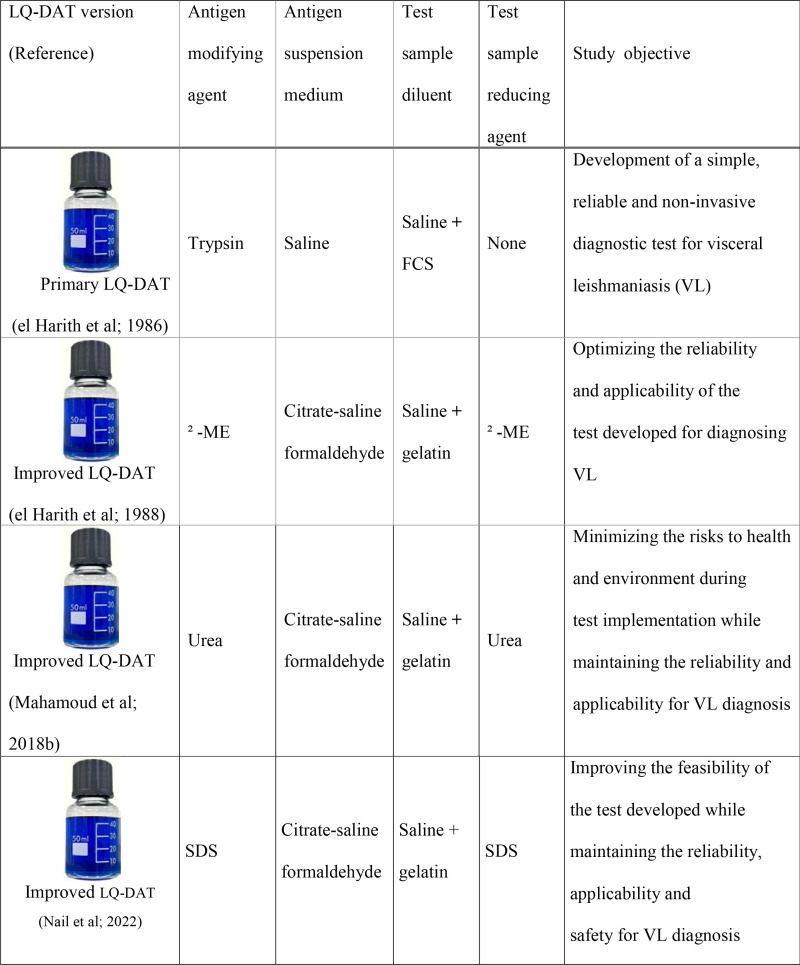
Evolutionary phases of the liquid direct agglutination test (LQ-DAT) for non-invasive detection of visceral leishmaniasis (VL).

**Set-up of Primary Liquid DAT (LQ-DAT):** A procedure based on the method developed by Allain and Kagan was followed, employing intact, trypsinized, and stained *L.(L.) donovani* MHOM/SD/00/LEM 1399 (Sudan) promastigotes to develop a primary LQ-DAT (P-DAT) [21,23]. To ensure feasibility for local production in endemic areas with suboptimal laboratory conditions, several improvements were introduced (Fig 1) [24–27].

Initially, the WHO-characterized *L. donovani* strain MHOM/SD/00/LEM 1399 was used for antigen processing. To improve reproducibility in VL-affected regions, uncharacterized strains isolated locally from confirmed VL cases in Sudan, Portugal, Brazil, and Bangladesh were later evaluated. Since antigen production requires large quantities of promastigotes, various axenic and synthetic culture media were assessed to optimize promastigote yields [24,25]. Across all tested media, cultures incubated at a constant temperature range of 20°C–26°C under shaking conditions consistently yielded higher promastigote concentrations than those maintained under static conditions.

Promastigote mass cultures, with densities of 4–5 × 10⁷/ml, were initiated in RPMI-1640 medium supplemented with HEPES, fetal calf serum (FCS), penicillin, and streptomycin. These cultures were harvested during the logarithmic growth phase (primarily elongated forms) and processed as described previously [23]. Following trypsin treatment and fixation with formaldehyde, promastigotes were stained with Coomassie Brilliant Blue (CBB) and resuspended at 1 × 10⁸/ml in a formaldehyde/normal saline solution.

To reduce the costs associated with promastigote mass culturing, attempts were made to lower their concentration per unit volume of suspension medium. Six antigen suspensions with promastigote concentrations ranging from 1 × 10⁸/ml to 1.4 × 10⁷/ml were evaluated to determine the minimum effective concentration. To prevent spontaneous agglutination and extend antigen shelf life, sodium citrate was used as an anti-clumping agent instead of normal saline [25,28]. The ready-to-use antigen was stored at 4°C for a period no longer than 75 days.

**Fig 2:**
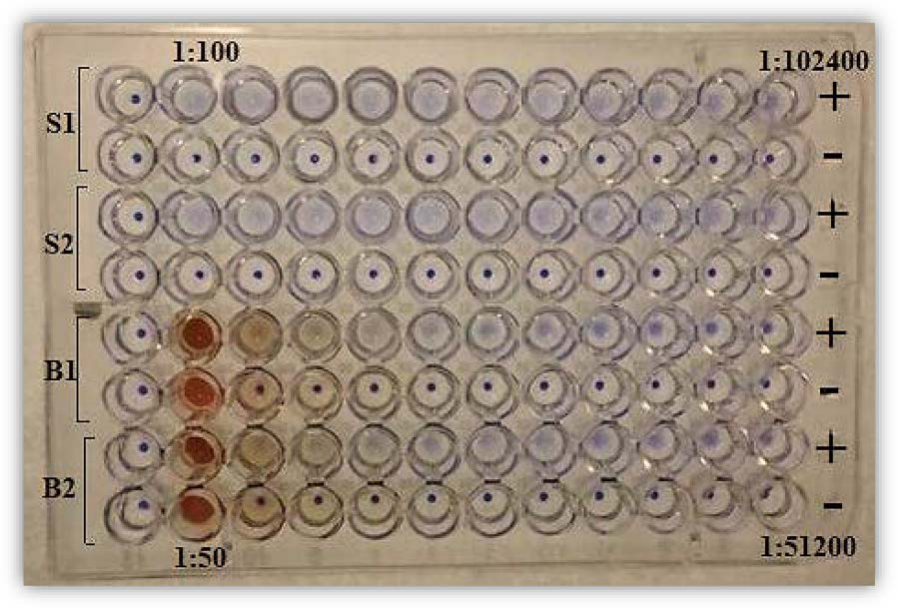
Procedural workflow of the liquid direct agglutination test (LQ-DAT) for mass screening of visceral leishmaniasis (VL).

**LQ-DAT Execution:** V-shaped well microtiter plates were used for test execution. Serum, plasma, or, in the case of children, anemic patients, or mass screening campaigns, whole blood samples spotted onto filter paper were serially diluted two-fold in normal saline supplemented with inactivated FCS or gelatin, starting from well #2. Well #1 served as the control, containing only the diluent. Test results were visually interpreted by identifying the dilution preceding the agglutination reaction endpoint, characterized by a sharp-edged blue spot (Fig 2) [23].

**LQ-DAT cutoff for VL:** To establish the cut-off titre for VL, sera from patients with confirmed disease (VL) and from individuals with non-VL conditions were analyzed. Further validation of this cut-off was conducted through blind testing of apparently healthy endemic individuals and patients with malaria, brucellosis, or splenomegaly of unknown etiologies residing in the Baringo VL-endemic district of Kenya [23].

The validity of the chosen cut-off was further confirmed by comparing it with the outcomes of the indirect immunofluorescence assay (IFAT) and enzyme-linked immunosorbent assay (ELISA) against disorders known to cross-react with VL, including AT and CD [29]. Reference VL sera were included as controls.

**Improving LQ-DAT Specificity:** To eliminate non-specific reactants and culture medium residues that resisted trypsin treatment and potentially caused false-positive reactions in patients with trypanosomiasis, alternative proteolytic and lipolytic agents were evaluated during antigen processing [25]. Promastigotes were processed following treatment with pronase, pancreatin, β-mercaptoethanol (β-ME), or lipase, as previously described for trypsin [23]. To further reduce test positivity due to potentially cross-reacting disorders, urea was incorporated to denature non-specific antibodies in serum or blood samples (Table 1, and Fig 1).

**Table 1:** Effect of β-mercaptoethanol (β-ME) on the performance of the primary direct agglutination test (P-DAT) using sera from patients with visceral leishmaniasis (VL) and from subjects with conditions potentially cross-reacting with VL.

Diagnosis	Number of sera tested	β-ME Treatment	Number of sera with indicated titre^a^:	
			≤ 1,600	≥ 3,200
Visceral leishmaniasis	36	– +	4 0	32 36
African trypanosomiasis	16	– +	13 14	3 2
American trypanosomiasis	29	– +	29 29	0 0
Auto-immune disorders	24	– +	23 24	1 0
Hematological Malignancies	71	– +	64 70	7 1
Other disorders^**b**^	92	– +	92 92	0 0
Apparently healthy endemic Kenyans	15	– +	8 15	7 0

a. VL cutoff titre: 3,200.

b. Including malaria and toxoplasmosis.

For more specific VL diagnosis, particularly in regions where CD is co-endemic, a LQ-DAT version analogous to VL-DAT was developed for CD [30,31]. The reliability of the CD-DAT was assessed in patients with confirmed CD, VL, AT, or other non-CD conditions.

Over 20 years of routine VL diagnosis in Sudan, patients occasionally presented with clinical features consistent with VL but exhibited titres in the high-negative or low-positive range (1:800–1:6,400). Unlike genuine VL cases, DAT titres in these patients remained unchanged despite several weeks of follow-up [27]. Information from physicians and guardians indicated outcomes such as mortality, failure to reach a final diagnosis, or leukemia occurrence. To exclude false positivity related to leukemia, further enhancements in LQ-DAT specificity were pursued. Despite the excellent performance achieved with β-ME and urea, sodium dodecyl sulfate (SDS) was evaluated as an additional sample denaturant (Figs 1 and 2) [27].

**Improving LQ-DAT Sensitivity** Encouraged by the effectiveness of exposing specific epitopes on the promastigote surface, β-mercaptoethanol (β-ME) was incorporated as a denaturant for the test serum or blood sample to enhance antigen binding in weakly reactive VL cases (Table 1 and Fig 1). Sera from 36 TAP-confirmed VL cases (treated 4–14 months earlier: eight with titres of 1:1,600–1:3,200 and 28 with titres >1:12,800) and from non-VL conditions, processed with or without β-ME, were tested against β-ME-processed antigens [25].

Building on previous findings with variable antigen types of *Trypanosoma gambiense*, we hypothesized that using antigens derived from *L. donovani* strains isolated locally could enhance agglutination against homologous sera from patients who contracted VL in the same or neighboring areas [32]. Antigen batches derived from *L. donovani* subspecies (*L. (L.) donovani* MHOM/SD/00/LEM 1399, *Leishmania (L.) infantum* MHOM/MA/00/ITMAP 263, and an uncharacterized Bengali *L. donovani* (*L.*) strain) isolated in Sudan, Morocco and Bangladesh, respectively, processed with β-ME, were tested against corresponding VL sera from TAP-confirmed Sudanese, Bangladeshi, and Moroccan VL patients [25].

**LQ-DAT Blind and Independent Evaluation:** The LQ-DAT was blindly applied to 280 inhabitants of the Perkerra area in Kenya and to 92 additional individuals with non-VL conditions [23].

To further assess the reliability of LQ- DAT for detecting VL throughout the course of infection, patients with either TAP-confirmed or TAP-unconfirmed diagnoses, likely representing early or mild infections, were included [6].

A ready-to-use LQ-DAT batch prepared locally at the Laboratory for Biomedical Research, University of Ahfad for Women, Omdurman, Sudan, was sent to the London Hospital for Tropical Diseases (LHTD) for independent evaluation [33]. This evaluation was conducted on 284 patients from 22 endemic countries.

**Application of LQ-DAT for Active VL Detection:** A cross-sectional study was conducted to detect active VL cases in the well-known endemic district of Mymensingh between September 1987 and November 1988 [34]. A total of 476,000 inhabitants were screened for fever through a house-to-house survey. VL suspicion was based on the presence of fever lasting more than four weeks, anemia, leukopenia, and a lack of response to anti-malarial treatment.

**Performance of DAT in a Major VL Outbreak:** The large-scale VL outbreak (1989–1991) in South Sudan posed significant health, social, and logistical challenges, necessitating the implementation of robust measures to contain the epidemic [35,36]. Following the correct identification of the “killing disease” by our team as VL and a timely response from the non-governmental organization *Médecins Sans Frontières* (MSF), LQ-DAT production was scaled up to provide sufficient quantities for screening thousands of suspected cases [36].

**Extending LQ-DAT Antigen Shelf-Life:** To prevent spontaneous promastigote aggregation and maintain antigen stability over extended periods, sodium citrate was incorporated into the suspension medium as anti-clumping agent (Fig 1) [25]. To ensure stability under high and fluctuating temperatures (23°C–47°C) characteristic of tropical endemic areas, the antigen was preserved using glycerol [37].

Antigen suspension prepared at 10 times the standard concentration was centrifuged to separate the pellet, which was resuspended in formaldehyde/citrate-saline with an equal volume of glycerol added [37]. To assess antigen shelf-life compared to the reference freeze-dried DAT (FD-DAT), an LQ-DAT batch preserved in 50% glycerol was tested. Both VL and non-VL sera were included in the evaluation. Additionally, the stability of the glycerol-preserved antigen was assessed after 13 months of storage at ambient temperatures (28°C–47°C) in the rural hospital of Doka, Eastern Sudan [38].

**Exclusion of Toxicants in LQ-DAT Execution:** Although the diagnostic reliability of LQ-DAT for VL is well-established, the use of toxicants such as β-mercaptoethanol (β-ME) and formaldehyde in antigen preservation and test execution poses significant risks in laboratories with limited safety infrastructure. Although β-ME is not classified as a carcinogen, repeated exposure to this toxic compound may cause liver and cardiac damage. Formaldehyde however is classified by the International Agency for Research on Cancer (IARC) as a potential carcinogen associated with nasopharyngeal cancer in humans. These hazards necessitate protective measures, such as fume chambers, which are often unavailable in resource-limited settings [26].

**Decentralizing Production of LQ-DAT:** In collaboration with central laboratories in Kenya, Bangladesh, Iran, Sudan, and Ethiopia, local production of LQ-DAT was successfully established in these major endemic regions. With support from TDR/WHO and the European Union, antigen batches derived from locally isolated *L. donovani* strains were independently produced by partner institutions [39–46].

**Results and discussion:** Except for patients with AT, all non-VL tested subjects exhibited titres <1:1,600, while all VL cases presented titres ≥1:3,200. Accordingly, 1:3,200 was established as the cut-off titre. Compared to spleen aspiration, the primary direct agglutination test (P-DAT) demonstrated 100% sensitivity and 99.3% specificity at this cut-off [23]. The validity of this threshold was further supported by IFAT and ELISA results. While titres remained unchanged in VL cases, positive readings were recorded in 8 (17%), 25 (55.6%), and 18 (40.0%) of the 45 trypanosomiasis sera tested using P-DAT, IFAT, and ELISA, respectively [29].

Among proteolytic agents other than trypsin, β-ME treatment of the promastigote antigen resulted in higher sensitivity and specificity than trypsin (Fig 1) [24]. Using β-ME-processed antigen, non-specific agglutination was significantly reduced for most non-VL conditions but remained detectable in some AT patients (Table 1) [24]. A marked reduction in false agglutination was also observed among patients with autoimmune disorders and hematological malignancies. However, complete inhibition of false agglutination was ultimately achieved by treating test sera with urea [25]. Following this modification, all sera from patients and experimentally infected animals with any of the seven most recognized *Trypanosoma* species tested unambiguously negative.

Sensitivity (95.6%) and specificity (94.7%) comparable to LQ-DAT for VL were achieved with LQ-DAT for CD. The simultaneous application of CD-DAT and VL-DAT is expected to be particularly valuable in Brazil, where both diseases are prevalent [31].

The validity of SDS incorporation as a test sample denaturant was confirmed through plasma testing from patients with six different types of hematological malignancies (HM) [27]. Compared to P-DAT (90%) and the rK39 rapid strip test (94%), SDS-DAT demonstrated superior specificity, achieving 100%.

Reported specificities for LQ-DAT in Sudan and other regions ranged from 78% to 100% when active visceral leishmaniasis (VL) cases were included. However, in one study where TAP-negative outcomes were used as the standard for VL exclusion, a lower specificity of 72% was reported [5]. In our opinion, a robust specificity criterion should include testing against known cross-reactive conditions or employing diagnostics with greater sensitivity than TAPs, such as polymerase chain reaction (PCR) or real-time qPCR. In contrast to this lower reported specificity, a 100% specificity was observed in nine of 49 patients who exhibited positive responses to treatment, as well as in 10 of 284 VL suspects from 22 endemic areas who were tested blindly or independently, with results matching those of culture or PCR outcomes [6,33].

The incorporation of sodium dodecyl sulfate (SDS) or β-ME as a sample denaturant effectively eliminated non-specific agglutination in patients with hematological malignancies or autoimmune disorders triggered by tumor cells or reduced tolerance to autoantigens [24,27]. Further research is required to determine whether similar autoimmune responses occur in trypanosomiasis or other cross-reactive conditions. Follow-up studies using SDS- or β-ME-treated DAT in ex-VL patients treated at different intervals could clarify the causes of “post-treatment positivity.” It is worth noting that resistance, relapse, or post-kala-azar dermal leishmaniasis (PKDL) in India and East Africa raises concerns regarding the effectiveness of first-line anti-leishmanial treatments in achieving a sterile cure. Sero-positive results were also reported in ex-VL Sudanese patients tested with an rK39-ELISA 24 months post-treatment [47]. Persistent seropositivity after treatment has been attributed to repeated sand fly bites or incomplete parasite clearance [48]. Elevated IgG1 levels post-treatment suggest a poor prognosis, and we hypothesize that a parallel increase in LQ-DAT titres and possibly rK39-ELISA absorbance values could indicate treatment failure or relapse [49].

A significant titre elevation was observed when using the matching serum/β-ME-treated antigen compared with trypsin-processed antigens derived from the same *L. donovani* strain against an untreated serum sample (Table 1). By combining the use of indigenous *L. donovani* subspecies from Sudan, Bangladesh, or Morocco and incorporating β-ME instead of trypsin during antigen processing, a threefold increase in titre was achieved in sera from VL-endemic areas [25]. This enhancement was evident even within localized VL foci [50]. For example, when tested against autochthonous antigen from Central Sudan, sera from VL cases in Umm Rimta exhibited significantly higher titres compared to those tested against non-autochthonous antigen from Gedarif in Eastern Sudan. Similarly, VL sera from Gedarif produced higher titres when tested against autochthonous antigens compared to non-autochthonous ones.

Excellent sensitivity values (>95%) for a freeze-dried DAT (FD-DAT) version were reported in a multi-center study conducted in East Africa (Ethiopia and Kenya) and the Indian subcontinent (India and Nepal) [51]. However, unlike FD-DAT, the rK39 strip test demonstrated appreciably lower sensitivity (<80%) in East Africa. Similarly, low sensitivity was reported for rK39 strip test in patients with clinical suspicion of VL in Sudan [52]. The relatively lower sensitivities (80.0%, 83.5%, 85.7%, and 88.1%) reported earlier with LQ-DAT emphasize the importance of using corresponding endemic *L. donovani* subspecies as antigens, ensuring proper transportation and storage of test sera, and minimizing the interval between specimen preparation and test execution. Sensitivity in some studies may also have been underestimated due to the inclusion of previously treated patients (6–36 months post-treatment) rather than active VL cases [24,29]. Notably, LQ-DAT titres in ex-VL patients treated 36 months earlier (1:1,600–1:3,200) were significantly lower than those treated four months prior (>1:12,800) [24]. As observed with LQ-DAT, Sudanese ex-VL patients exhibited similar positive outcomes when tested with rK39 ELISA 24 months after successful treatment [47]. These findings provide valuable insights for evaluating treatment response and identifying potential cases of relapse or incomplete parasite clearance.

The diagnostic efficiency of LQ-DAT in VL populations with immunodeficiencies or suppressed immunity remains a challenge [53,54]. In an Ethiopian VL-endemic population of 699 individuals with an HIV prevalence of 34%, LQ-DAT demonstrated higher sensitivity (89%–95%) compared to the rK39 reference test (84%–87%). Combining DAT with rK39 further increased diagnostic reliability to 98%–99%, irrespective of HIV status [55]. In another Ethiopian cohort with a higher HIV prevalence (50.7%–56.0%), nearly identical sensitivity (97.7%) was reported for LQ-DAT. Only 2 of 51 VL/HIV co-infected patients and none of the 40 VL-only patients tested negative [56]. Comparable LQ-DAT performance was observed in a French cohort of 76 individuals, including 10 HIV-positive cases, when tested with homologous (*L. infantum*) or standard heterologous (*L. donovani*) antigens [57]. Lowering the VL cut-off titre (1:640 vs. 1:3,200) had no discernible impact on test specificity [57,58]. However, omitting β-ME as a denaturant in the sample diluent significantly reduced sensitivity in HIV-VL co-infected patients (54.5%) compared to VL-only patients (88.5%) [59]. Although sensitivity was lower in HIV-positive patients, no significant differences in FD-DAT titre levels were observed between HIV-positive and HIV-negative groups [55].

A large study conducted in India (7,950 participants) and Nepal (5,336 participants) demonstrated a strong correlation between positive DAT or rK39-ELISA results and VL progression to clinical stages, highlighting their ability to detect VL before the onset of full-blown disease [60]. Similar promising results have been reported using the LQ-DAT with a combination of recombinant rK26 and rK39 antigens for the detection of anti-*Leishmania infantum* antibodies in both human and reservoir hosts in Iran [61]. In a study of 1,606 individuals from 27 endemic villages in Bihar, India, qPCR and rK39-ELISA were recommended as supplementary tools to support FD-DAT-positive outcomes and guide prophylactic treatment [62]. The specificity and sensitivity of DAT and PCR were further demonstrated by Bhattarai et al. (2009), who identified asymptomatic *Leishmania* infections among healthy Nepalese individuals living in a VL-endemic region [63].

Upon the blind testing of sera from the endemic population in the Perkera area of Kenya, it was found that among the seven individuals who tested at titres >1:1,600, two had positive spleen aspiration results, three had been treated and were apparently cured, one exhibited typical VL symptoms but had a negative spleen aspirate, and one appeared healthy with a normal spleen size [23].

Of 49 Sudanese patients previously treated for VL at Hawata Rural Hospital (Kassala Province, Sudan) and later blindly tested with LQ-DAT, 40 had both positive bone marrow aspirates and LQ-DAT results and responded positively to specific VL treatment [6]. Nine patients (18.4%) with negative bone marrow aspirates but positive LQ-DAT titres demonstrated a favorable response to first-line anti-leishmanial therapy.

The independent evaluation of LQ-DAT conducted at the London Hospital for Tropical Diseases (LHTD) on 284 patients from 22 endemic countries indicated that all 10 suspected VL cases tested positive using the Sudan-prepared LQ-DAT antigen. These cases also demonstrated 100% concordance with an LHTD-prepared LQ-DAT antigen, TAP, PCR, or specific treatment outcomes [33]. This high specificity of LQ-DAT was further confirmed by the performance of FD-DAT (99.2%) when tested in VL-suspected populations from two endemic regions in Sudan [52]. These findings, along with results from earlier studies in Kenya, Sudan, and Bangladesh, provide compelling evidence that LQ-DAT is both highly sensitive and specific for detecting VL in both early stages, before amastigotes become visible, and in advanced stages when amastigotes are present in tissue aspirates.

Among the 476,000 inhabitants screened for VL symptoms in the endemic district of Mymensingh, Bangladesh, 1,273 individuals tested positive with LQ-DAT, of whom 715 (56.2%) had both positive bone marrow aspirates and LQ-DAT results. The remaining 558 individuals (43.8%) had negative TAP results but tested positive with LQ-DAT (Table 2). As observed with the 715 confirmed cases, a positive response to first-line anti-leishmanial sodium stibogluconate treatment was evident in the vast majority (547, 98.0%) of the TAP-negative cases [34]. This finding suggests that withholding treatment based solely on negative microscopy results would have led to an unacceptably high mortality rate (43.8%).

**Table 2:** Response to specific anti-leishmanial treatment in 473 suspected cases identified as non-visceral leishmaniasis (non-VL) by tissue aspiration procedures (TAP) but as visceral leishmaniasis (VL) cases by the direct agglutination test (DAT) during routine, endemic, or epidemic investigations conducted by the principal investigators and counterparts between 1989 and 2007.

Authors	Type of VL study	Endemic area (population)	No of suspects	Tissue aspiration procedure ^a^	Final VL diagnosis/treatment ^b^		
					+MIC	+ DAT	Response to specific treatment (no of cases)
Abdel-Hameed et al; 1989 [6]	Routine diagnosis	Central Sudan (203)	49	BM and LN	40	49	Favorable (49)
Chowhury et al; 1991 [40]	Epidemiologic	Bangladesh (1273)	715	BM	558	715	Favorable (715)
De Beer et al; 1991 [42]	VL outbreak	South Sudan (2714)	1195	BM and LN	325	513	Favorable (509) ^**c**^
Chowhury et al; 1993 [54]	Epidemiologic	Bangladesh (918)	379	BM	29	125	Favorable (125)
el Mutasim et al; 2006 [45]	Routine diagnois	Eastern Sudan (308)	115	LN, BM and SA	105	115	Favorable (115)
Mansour et al; 2007 [38]	Epidemiologic	Eastern Sudan (416)	322	LN	125	142	Favorable (142)

a. Tissue aspiration procedures were conducted for amastigote demonstration in lymph node (LN), bone marrow (BM), or spleen (SA) aspirates.

b. Final VL diagnosis was established either by positive microscopy (+MIC), symptoms with a positive DAT result (+DAT) and favorable response to specific treatment.

c. Four patients died before, during, or after treatment.

Although the exact timeframe for LQ-DAT to signal VL is not yet fully determined, the steady increase in test titre levels observed during follow-up of individuals who initially tested at high-negative (1:400) or marginal (1:800–1:1,600) titres suggests an interval of 5–8 weeks. The characteristic immediate positive response to therapy was reflected in the resolution of fever and regression of spleen size within 5–7 days post-treatment [36].

Among 1,195 individuals identified as VL suspects from the displaced Nuer Tribe population (2,714) in Khartoum (Sudan), 513 tested positive using LQ-DAT. Of these, 325 (63.4%) also had positive TAP outcomes [36]. Given the high diagnostic reliability of LQ-DAT observed in Northern Sudan and Bangladesh, and the risks of withholding treatment, first-line anti-leishmanial therapy (Pentostam) was administered to all 513 suspects, including the 188 individuals (36.6%) with negative TAP outcomes. Of the 513 treated individuals, 509 (99.2%) responded favorably, while 4 (0.6%) passed away before or during treatment. It is suspected that a significant proportion of the 188 TAP-negative individuals had mild or early VL infections that evaded microscopic detection due to low amastigote densities in organ aspirates.

Despite the high prevalence of intercurrent infectious and non-infectious disorders (e.g., upper respiratory infections, pneumonia, malaria, tuberculosis, and malnutrition) among the displaced population, no hindrance to treatment response was observed. Although HIV screening was not conducted, no resistance to VL chemotherapy was detected. Timely intervention, particularly in unconfirmed cases, likely contributed to the remarkably low mortality rate (0.6%).

Regardless of storage temperature, titres obtained with the LQ-DAT antigen preserved in 50% (v/v) glycerol against VL and non-VL sera were concordant with those of the standard reagent stored at 4°C. Differently than with the primary DAT (P-DAT) (75 days) the glycerol-preserved antigen remained stable for up to 12–13 months at constant (22°C–37°C) or variable ambient (28°C–47°C) temperatures in Iran and Eastern Sudan, respectively [38,63]. Contradicting earlier reports, freeze-drying of the antigen in DAT failed to maintain stability at temperatures >35°C [28,64,65]. Our efforts to enhance the stability and feasibility of the freeze-dried DAT version (FD-DAT) were successful through the replacement of the original antigen suspension medium (normal saline) with the anti-clumping agent formaldehyde citrate saline (FCS) [28]. Additionally, replacing β-ME with the non-toxic or minimally toxic agents urea and sodium dodecyl sulfate (SDS) mitigated risks for users and the environment, further encouraging LQ-DAT production in laboratories with minimal or no safety facilities [26].

In collaboration with central laboratories in Kenya, Bangladesh, Iran, Sudan, and Ethiopia, local production of LQ-DAT was successfully established in these major endemic regions. With support from TDR/WHO and the European Union, antigen batches derived from locally isolated *L. donovani* strains were independently produced by partner institutions (Table 3) [39–46].

**Table 3:** Diagnostic accuracy of the direct agglutination test (DAT) versions compared to outcome of tissue aspiration procedures (TAP) in studies conducted by the principal investigators and counterparts between 1986 and 2023.

Authors (VL endemic region/country)	VL cases in study population	LQ-DAT version^a^	DAT diagnostic accuracy based on TAP outcome:	
			Sensitivity	Specificity
**EAST AFRICA:**				
el Harith et al, 1986 (Kenya) [17]	31 (385)	P-DAT	83.9%^**b**^	79.5%^**b**^
el Harith et al, 1987 (Kenya) [23]	27 (400)	P-DAT	80.0%^**c**^	72.6%^**c**^
Kager et al; 1989 (Kenya) [55]	5 (280)	P-DAT	100.0%	99.3%
Abdel-Hameed et al 1989 (Sudan) [6]	40 (403)	β-ME DAT	100.0%	100.0%
de Beer et al. 1991 (Sudan) [42]	325 (1195)	β-ME DAT	100.0%	100.0%
el Harith et al, 2003 (Sudan) [43]	24 (78)	β-ME DAT	100.0%	100.0%
Abass et al, 2006 (Sudan) [47]	40 (198)	β-ME DAT	100.0%	98.8%
el Mutasim et al.2006 (Sudan) [45]	195 (308)	β-ME DAT	86.7%	92.1%
Abass et al, 2007 (Sudan) [46]	17 (48)	β-ME DAT	100.0%	100.0%
Mansour et al., 2007 (Sudan) [59]	125 (416)	β-ME DAT	100.0%	100.0%
Osman et al, 2016 (Sudan) [51]	96 (138)	β-ME DAT	99.0%	100.0%
Mahmoud et al, 2018a (Sudan) [20]	177 (563)	Urea-DAT	100.0%	100.0%
Mahmoud et al, 2018b (Sudan) [30]	46 (66)	β-ME DAT	100.0%	100.0%
Nail et al, 2023 (Sudan) [21]	18 (88)	SDS DAT	100.0%	100.0%
**INDIAN SUB-CONTINENT**				
Chowdhury et al; 1991 (Bangladesh) [40]	558 (1273)	β-ME DAT	100.0%	100.0%
Chowdhury et al; 1993 (Bangladesh) [54]	125 (918)	β-ME DAT	100.0%	100.0%
Al Masum, et al. 1995 (Bangladesh) [50]	257 (480)	β-ME DAT	100.0%	100.0%
**GLOBALLY**				
Andrade et al; 1987 (Brazil) [25]	14 (265)	P-DAT	100.0%	100.0%
el Harith et al, 1988 (Kenya, Brazil and India) [18]	62 (808)	β-ME DAT	93.5% ^**d**^	97.8% ^**d**^
de Korte et al, 1990 (France) [36]	32 (38)	β-ME DAT	100.0%	100.0%
el Harith et al, 2019 (22 endemic areas) [26]	10 (284)	β-ME DAT	100.0%^**e**^	100.0%^**e**^

a. Sera, plasma or whole blood samples tested with trypsin-, β-ME-, urea- or SDS- DAT versions.

b. Sera from 31 VL cases, including 10 from patients treated 3–36 months earlier and from 27 patients with African trypanosomiasis.

**c.** Sera from 27 VL cases, including 10 from patients treated 4–14 months earlier and from 45 patients with African or American trypanosomiasis.

d. Sera from 62 VL cases, including 10 collected during follow-up or after treatment (1–14 months), and from 45 patients with African or American trypanosomiasis.

e. VL suspects from 22 countries evaluated independently at the London Hospital for Tropical Diseases.

**LQ-DAT Limitations:** In comparison with the WHO rK39 rapid reference test (10–15 minutes), obtaining a qualitative provisional outcome with LQ-DAT requires 2–3 hours. For a possible semi-quantitative assessment of VL severity, an incubation period of 8–12 hours is necessary. Likely due to the freeze-drying effect, significantly longer incubation periods (5–7 hours) are required for a provisional outcome with the second WHO reference test (FD-DAT) [24,28,66].

As with both rK39 and FD-DAT reference diagnostics, LQ-DAT lacks the ability to qualitatively distinguish between active and past VL infections. However, a consistent and steady reduction in LQ-DAT titre levels was observed in successfully treated VL cases [24,25]. Whether this phenomenon could be advantageous in assessing treatment effectiveness requires further follow-up studies.

Comparable to the two reference diagnostics, a noticeable reduction in anti-*L. donovani* antibodies was observed with LQ-DAT in VL-HIV co-infected patients. Possibly due to its relatively higher sensitivity (89%–95%) compared to the reference rK39 test (84%–87%), LQ-DAT successfully identified VL cases in an Ethiopian population with an HIV prevalence rate of up to 56.0% [56]. Despite PCR application, a slightly higher proportion (97%) of VL/HIV co-infected patients was identified [19]. We anticipate that incorporating corresponding endemic *L. donovani* isolates as antigens in LQ-DAT may compensate for the 2%–5% loss in sensitivity in areas with high HIV/VL co-endemicity. Further rigorous evaluation in patients with full-blown or even higher HIV prevalence rates is necessary to determine LQ-DAT’s efficacy relative to existing molecular methods.

**Conclusions and Recommendations:** Despite its unknown chemical composition and mono- or multi-epitope nature, the trypsin- or β-mercaptoethanol (β-ME)-treated *L. donovani* promastigote antigen has demonstrated high efficacy for VL diagnosis, regardless of the subspecies (*L. (L.) donovan or, L. (L.) infantum*) or strain (Table 3). However, relatively higher sensitivities were observed when using corresponding endemic subspecies or autochthonous strains of *L. donovani*. Whether the evaluated cleaving agents unfold surface glycoproteins to expose specific reactive carbohydrate epitopes or remove non-specific epitopes remains unclear and warrants further investigation [25,67].

Active surveillance aimed at identifying VL cases before the onset of full-blown clinical symptoms – while also improving cure rates – can also help mitigate the risks of resistance, relapse, and post-kala-azar dermal leishmaniasis (PKDL). Achieving this requires a diagnostic tool that is feasible, reliable, and applicable in resource-limited settings. Tools such as LQ-DAT have proven effective in mass screening campaigns, including those conducted in Bangladesh’s Mymensingh district and among displaced individuals from South Sudan’s Nuer Tribe [34,35].

Beyond its diagnostic reliability, LQ-DAT offers significant practical advantages for resource-limited laboratories in the VL affected areas. Unlike molecular techniques, the high potential for local LQ-DAT production ensures sustainable application [17–19,41,68]. By adhering to strict test processing guidelines—described here or elsewhere—valid LQ-DAT batches can be successfully produced with financial support from ministries of health, universities, WHO, or humanitarian organizations. Test batches for large-scale screening (75,000–100,000 individuals) can be dispatched to affected areas within 15–20 days without requiring cold-chain logistics [34,35].

Unlike other serodiagnostic methods, LQ-DAT execution does not require specialized equipment to separate serum or plasma. Due to its minimally invasive nature, utilizing finger-prick blood sampling (Fig 2), no resistance or objections were encountered from VL suspects or health authorities in Bangladesh or Sudan during active detection and intervention efforts in South Sudan. LQ-DAT performs reliably across a broad temperature range (20°C–47°C), and its sharp-edged blue spot reaction endpoint simplifies titre determination compared to the subjective interpretation of rK39 band intensities (Fig 2) [69]. The exclusion of formaldehyde and β-ME enhances the test’s safety for both users and the environment [26]. With 35 years of demonstrated stability under highly fluctuating temperatures (4°C–47°C) across multiple regions, LQ-DAT holds strong potential for routine and large-scale application worldwide.

### Key learning points

- Patients presenting with typical VL clinical signs and high-negative titres (1:400–1:1,600) should be weekly or biweekly undergo follow-up with LQ-DAT.
- If there is a steady increase in DAT titre levels to ≥1:3,200, and malaria, typhoid, or tuberculosis have been excluded, VL should be seriously considered.
- If LQ-DAT titre levels remain unchanged after 4–6 weeks of follow-up and trypanosomiasis has been excluded, consultation with a hematologist or oncologist is recommended.

### Top five papers

- el Harith A, Kolk AHJ, Leeuwenburg J, Muigai R, Huigen E, et al.. Improvement of a direct agglutination test for field studies of visceral leishmaniasis. *J Clin Microbiol*. 1988; 26, 1321–1325.
- Hasker E, Malaviya P, Gidwani K, Picado A, Ostyn B, et al.. Strong association between serological status and probability of progression to clinical visceral leishmaniasis in prospective cohort studies in India and Nepal. PLOS Negl Trop Dis. 2014; https://doi.org/10.1371/journal.pntd.0002657.t004.
- Boelaert M, Bhattacharya S, Chappuis F, Elsafi SH, Hailu A, et al.. Evaluation of rapid diagnostic tests: visceral leishmaniasis. Nat Rev Microbiol. 2007; S30–S39. https://doi.org/10.1038/nrmicro1766.
- de Beer P, el Harith A, Deng LL, Semiao-Santos SJ, Chantal B, et al.. A killing disease epidemic among a displaced Sudanese population identified as visceral leishmaniasis. Am J Trop Med Hyg. 1991; 44, 283–289.
- Chowdhury S, Haque F, Al-Masum A, el Harith A, Karim E.. Positive response to sodium antimony gluconate in visceral leishmaniasis sero-positive patients. Am J Trop Med Hyg. 1991;44, 390–393.
